# The Role of Proanthocyanidins Complex in Structure and Nutrition Interaction in Alfalfa Forage

**DOI:** 10.3390/ijms17050793

**Published:** 2016-05-23

**Authors:** Arjan Jonker, Peiqiang Yu

**Affiliations:** 1 College of Agriculture and Bioresources, University of Saskatchewan, 51 Campus Drive, Saskatoon, SK S7N 5A8, Canada; 2 Current address: Grasslands Research Centre, AgResearch Ltd., Tennent Drive, Private Bag 11008, Palmerston North 4442, New Zealand

**Keywords:** proanthocyanidin, alfalfa, molecular structure and nutrition interaction

## Abstract

Alfalfa (*Medicago sativa* L.) is one of the main forages grown in the world. Alfalfa is a winter hardy, drought tolerant, N-fixing legume with a good longevity, high yield, high nutrient levels, high digestibility, unique structural to non-structural components ratio, high dry matter intake, and high animal productivity per hectare. However, its main limitation is its excessively rapid initial rate of protein degradation in the rumen, which results in pasture bloat and inefficient use of protein with consequent excessive excretions of nitrogen into the environment. Proanthocyanidins are secondary plant metabolites that can bind with protein and thereby reduce the rate and extent of ruminal protein degradation. However, these secondary metabolites do not accumulate in alfalfa. This review aims to firstly describe the events involved in the rapid release of protein from alfalfa and its effect on ruminant nutrition, environmental pollution, and pasture bloat; secondly, to describe occurrence, structure, functions and benefits of moderate amounts of proanthocyanidin; and finally, to describe the development of alfalfa which accumulates moderate amounts of proanthocyanidins. The emphasis of this review focuses on the role of proanthocyanidins compounds in structure and nutrition interaction in ruminant livestock systems.

## 1. Mechanisms for the Release of Protein from Model Alfalfa

Alfalfa has an excessively rapid initial rate of protein degradation compared to grasses and many legumes like cicer milkvetch (*Astragalus cicer* L.), sainfoin (*Onobrychis viciifolia* L.), birdsfoot trefoil (*Lotus corniculatus*. L.), big trefoil (*Lotus pedunculatus* L. also known as *Lotus major* L. and *Lotus uliginosus* L.) and sulla (*Hedysarium coronarium* L.) but similar to white clover (*Trifolium repens* L.; also known as ladino clover), red clover (*Trifolium pratense*. L) and wheat pasture forage (*Triticum aestivum*) [1,2,3,4,5,6,7,8,9,10,11,12,13]. The main portion of forage proteins is located in leaf mesophyll cells. Soluble proteins can be divided into fraction I proteins (Ribulose-1,5-*bis*phosphate Carboxylase (Rubisco)) which makes up 30%–50% of soluble protein, fraction II protein (several proteins present in small amounts, e.g., plant enzymes and hormones) and chloroplast membrane proteins [14]. On a plant cell level, rapidly degradable nitrogen (N) fractions in plants are non-protein N (NPN; e.g., NH_4_^+^ and NO_3_^−^, which are mainly found in the plant cell vacuole) and cytoplasmic and soluble protein (mainly Rubisco in alfalfa, which is mainly found in the plant cell chloroplast) [15]. The first mechanism by which these soluble protein fractions are released from the plant cell is ingestive chewing during grazing by livestock. Chewing cracks the cuticle and plasma membrane, destroys mesophyll cells and reduces the forage particle size. During chewing, the N components from the vacuoles were found to be released at a faster rate and to a larger extent than N from the chloroplasts [15,16]. White clover was found to have a faster release of the total cell content and intracellular N from the plant cell after ingestive chewing (before entering the rumen) than three grass species [17] and alfalfa and red clover had a faster release of soluble proteins after ingestive chewing than sainfoin (Table 1) [18]. Epidermal and mesophyll cells of alfalfa and white and red clover were found to be thinner and less resistant to mechanical rupture than those of birdsfoot trefoil, sainfoin and cicer milkvetch (Table 1) [19,20,21]. Waghorn *et al.* [22] found that ~40% of perennial ryegrass and ~20% of alfalfa plant cells reached the rumen intact after ingestion, while in general it is believed that over 50% of plant cells reach the rumen intact [23].

Some plant cells are still intact and metabolically active when entering the rumen. The conditions in the rumen (temperature, anaerobic nature and microbial colonization) cause a stress reaction in the viable plant cells and result in a release of protein, due to in plant metabolic (e.g., proteinase) activity [23,28]. Proteolytic activity indexes of fresh plant tissue incubated over a gelatin substrate gel was high for *Medicago* species (mean 0.56, for alfalfa 0.77) and *Avena* (oat 0.71) but low for the *Trifolium* (with clover 0.24, red clover 0.17), *Triticum* (wheat 0.08), *Lolium* (perennial ryegrass 0.22) and *Lotus* (birdsfoot trefoil 0.14, big trefoil 0.16) species (Table 1) [27]. Thus, it seems that forages with high ruminal protein degradation do not necessarily have higher proteolytic activity. Kingston-Smith *et al.* [24] found that ions which are stored in the vacuole (e.g., Ca^2+^, Mg^2+^, NH_4_^+^ and NO_3_^−^) are released when fresh plant material was incubated in H_2_O at 39 °C (rumen temperature) without microbes (Table 1). This indicates that membranes become weaker and more permeable over time. Probably, absorption of H_2_O into the plant tissue is involved (cells swell and burst) and allows small molecules including ions, soluble carbohydrates and small proteins to escape from the plant cell [24]. The rate at which ions were released was fast for white and red clover and sainfoin, slow for birdsfoot trefoil and intermediate for alfalfa [24].

Intact plant cells have to be ruptured before microbes can enter the cell [29]. Many of these plant cells will be ruptured during re-mastication (rumination) of the cud by the ruminant [16]. When new plant material enters the rumen, microbes will attach to it within five minutes [30]. Microbes will try to invade the plant cell through the stomatal cavity, lenticels and disrupted cells, after which intracellular micro-colonies are formed that can easily disrupt and digest mesophyll cells from the inside out [29,30]. The time needed to disrupt leaf tissue and to invade and digest mesophyll cells was shorter for alfalfa, and white and red clover than for birdsfoot trefoil, sainfoin and cicer milkvetch (Table 1) [26,29,31]. The breakdown of plant particles in the rumen was found to be faster for alfalfa than for perennial ryegrass (*Lolium perrene* L.) [22].

Overall, alfalfa protein was found to have a higher solubility, faster degradation rate and larger extent of ruminal degradation than protein of sainfoin and grasses [8,32]. Besides having plant cells that are more resistant to rupture, legumes like sainfoin, birdsfoot trefoil, big trefoil and sulla contain proanthocyanidins which reduce protein degradation and availability in the rumen [9]. However, proanthocyanidins are not synthesized in the aerial parts of alfalfa and are therefore not present in their forage [10].

However, alfalfa remains popular because it has higher seed germination, higher pasture longevity and higher grazing tolerance compared with legumes like sainfoin, birdsfoot trefoil and cicer milkvetch [33]; compared with grasses, alfalfa has a higher animal dry matter (DM) intake and requires lower fertilizer inputs [5]; and compared with other legumes and grasses, alfalfa has a higher DM yield and animal production per hectare [3,34].

## 2. Protein Degradation, Nitrogen to Energy Synchronization Balance and Nitrogen Excretion 

The amino acids that are absorbed in the small intestine of ruminants originate from intestinal digested microbial bodies (protein), intestinal digested dietary protein that escaped rumen microbial degradation and endogenous protein [35]. Microbial-N that enters the small intestine ranges from 60% to up to 90% of total N entering the intestine [36]. For their growth, rumen microbes require N, energy and essential minerals. Carbohydrates are the main source of energy used by microbes [37,38]. Optimal ratio between ruminal available N and energy required for microbial growth was found to be approximately 25 g·N/kg DM degraded in the rumen [39], 25 g·N/kg organic matter (OM) degraded in the rumen [40] or 32 g·N/kg carbohydrate (CHO) degraded in the rumen [41,42].

In freshly harvested alfalfa at different stages of growth, rumen degradable N:DM ratio ranged from 31–49 g·N/kg DM with extent of ruminal DM degradation ranging from 55% to 80% which is lower than the range of 76% to 90% for extent of ruminal crude protein (CP) degradation (Table 2). NRC [43] recommends that a total diet consists for 14%–18% of CP with 62%–65% of CP consisting of rumen degradable CP, while Broderick *et al.* [36] found (analysis of 32 studies with 122 diets) dietary CP of 15% from which 70% CP in the rumen degradable form to be optimum. The forage CP content, rumen degradable CP and imbalance between ruminal degradable N and DM tends to decrease with advancing plant maturity stages (Table 2).

The rate and extent of alfalfa protein degradation are not only excessive, but the supplied amount of rumen available energy from alfalfa is also not sufficient to support optimal conditions for microbial growth. Degradation of fibrous carbohydrates is slower in forages than for non-fiber carbohydrates (NFC). For alfalfa at three stages of growth (pre/early-bud, late-bud and early flower stages) fractional degradation rate of neutral detergent fiber (NDF) ranged from 3%–8%/h which is slower than the range of 7.8%–16%/h found for NFC in two studies [8,48]. The CP properties of alfalfa improve with advancing maturity, but NDF content will increase as well, which counterbalances the improved CP properties in a negative fashion. Therefore, the imbalance between the release of N and energy in the rumen can be decreased by decreasing protein content, solubility or degradation rate and/or increasing content, solubility or degradation rate of carbohydrates.

The excess protein released into the rumen above microbial requirements is mainly deaminated into ammonia (NH_3_), energy and volatile fatty acids (mainly propionate and branch chain fatty acids) by rumen microbes [49,50]. The energy is used for microbial growth and excess ruminal NH_3_ is absorbed across the rumen wall and converted in the liver mainly into urea at the cost of energy [51,52]. Urea-N can re-enter the rumen via secreted saliva or by direct diffusion/active transport from blood into the rumen where it is converted back into NH_3_ by ureolytic bacteria [53,54]. When sufficient energy is available, NH_3_ is used for microbial protein synthesis; otherwise when energy is insufficient, NH_3_ will be mainly lost to the animal and excreted via urea in the urine [51,55]. At different levels of protein intake from total mixed rations with different levels of N and ruminal N degradability and different forage:concentrate ratios, urine-N excretion ranges from 133–592 g/kg·N intake [56]. Excreted urinary urea-N is easily volatilized to NH_4_ or converted to NO_3_^−^ which contribute to environmental acidification and eutrophication, respectively [51,56]. In addition to these environmental drawbacks, high blood NH_3_ and urea levels also have a negative effect on reproduction and fertility of cattle [52].

Valkeners *et al.* [57] found that an unsynchronized N to energy supply during parts of the day can be balanced by urea-N recycling when the overall N to energy supply is balanced on a daily basis. Some recent research suggests that diets with oscillating dietary protein (unsynchronized but balanced) are utilized more efficiently, probably because of increased utilization of recycled urea-N into microbial cells [58,59,60]. However, as described before, for alfalfa the release of excess N relative to energy in the rumen is both unsynchronized and unbalanced and will lead to an inefficient use of dietary N by the animal and excessive excretion of N into the environment. Conversion of protein into energy results not only in protein losses for the animal but it also yields less energy (13.6 mol·ATP/kg CP) than derived from fermentation of carbohydrates (23.9–27.3 mol·ATP/kg CHO) for microbial growth [49,61]. Moreover, excessive ruminal protein degradation results in a reduced portion of dietary protein escaping to the lower digestive tract, a process that is required for optimum animal performance of high producing cattle [62,63].

## 3. Alfalfa and Pasture Bloat

Pasture bloat arises from rumen fermentation gases, which become trapped in a viscous stable protein foam that prevents normal eructation of microbial fermentation gasses by the animal [64,65]. The accumulation of gas in the rumen causes dissention of the rumen and exerts pressure on organs like the lungs, heart and vagus nerve. This can lead to the death of the animal under severe conditions [66,67]. Even considering the relatively low number of animals grazed on alfalfa in North America, cattle mortality due to pasture bloat was estimated to be as high as 1.5% with economic losses estimated at $125 million a year [68,69].

Rumen conditions that favor the formation of stable protein foams include the nature of the surface active materials at the gas–liquid interface provided by the rumen environment (pH, viscosity, ionic composition, soluble protein concentration, and microbial species composition) and adequate gas production [33,70]. Table 3 shows some rumen and animal characteristics of cattle prone to pasture bloat compared with animals that do not bloat on the same pasture.

Increased viscosity of the rumen liquid may be due to increased protein, carbohydrate, and nucleic acid concentrations [79], viscous saliva [73] and slime produced by certain microbes [80,82]. The increased viscosity of rumen liquid makes it more difficult for feed particles to move within the liquid and increases particle matter buoyancy with consequent increased particle retention (chlorophyll) in the rumen [71,75,83]. The viscous film at the gas–liquid interface can be harvested by methanol precipitation. This film of bloating cattle on legume pastures (white clover) had a CP and CHO composition (63% and 15%, respectively) similar to foam from a bloating animal [79,84].

Because positive correlations between pasture bloat and alfalfa soluble protein content have been reported frequently, it is generally accepted that rapid initial release of protein from alfalfa is involved in its propensity to cause pasture bloat [14,72,85]. Soluble proteins, which are rapidly released into the rumen liquid, are thought to form a viscous film at the gas–liquid interface and this could act as an active agent for the formation of foam that might result in frothy bloat [72,80]. The proteolytic bacterial strain *Streptococcus bovis* (*S. bovis*) cultured on a protein extract (from wheat forage) was found to have a high growth rate and to produce large amounts of slime [80]. Proteolytic microbial populations in the rumen of cattle grazing perennial ryegrass/white clover pasture were found to consist of 61% *S. bovis* like strains [86]. Thus, part of the viscous film formed at the gas–liquid interface might originate from protein degrading microbes that produce a viscous slime. However, *in vitro* foam stability of aqueous leaf extracts was previously found to be related to *in vitro* foam stability from rumen liquid of bloating animals and to *in vivo* bloat incidence in cattle [87,88]. This suggests that slime produced by microbes are not required for the formation of a viscous film at the gas–liquid interface and thus not required for the formation of stable foam in the rumen.

Incidence of bloat in grazing cattle is higher when offering alfalfa at a vegetative stage than at advanced stages of maturity [89]. Vegetative alfalfa has a higher leaf to stem ratio, higher protein content and higher digestibility compared with more mature alfalfa [90,91]. In general, pasture bloat occurs within two to three hours after moving cattle to a bloat inducing pasture [33,92]. Cattle graze the pasture canopy from the top to the bottom of the plants removing around 35% of the sward height per bite [93]. The top of the canopy has a higher leaf to stem ratio than the bottom of the canopy [91]. So, at the initial stage of grazing cattle consume mainly leaves. Leaves have a higher soluble protein content, higher ruminal digestibility, lower fiber content and higher foaming properties compared with stems [90,94,95]. Thus the higher soluble protein content and digestibility in leaves and vegetative growth might explain the higher bloat incidence shortly after allocating cattle to a new pasture or to a vegetative pasture. However, the protein content in rumen liquid of bloating cattle was not different than that of non-bloating cattle on the same pasture. This suggests that other components are involved in the stabilization of bloat related foam [68,75,81].

Alfalfa leaves and younger vegetative growth were found to have a higher saponin content compared with stems and more mature growth [96]. Saponins form a stable froth when shaken in water. When saponins are released into the rumen liquid, they reduce smooth muscle activity and thereby reduce rumen motility. This reduces the rumen digesta passage rate and may directly reduce or stop the eructation reflex [97,98]. Saponins released in the rumen liquid are extensively degraded by saponin degrading microbes (e.g., *Butyrivibrio*) which were found to produce a viscous slime [82]. However, cattle grazing a high saponin (1.9%) alfalfa cultivar were no more prone to pasture bloat than cattle grazing a low saponin (0.8%) cultivar, and administration of alfalfa juice with saponins to cattle and sheep did not produce bloat [99,100]. Besides, bloat occurs on wheat pastures as well and wheat forage does not contain saponins [101,102]. However, some reported studies detected bloat signals like distention of the rumen and animal distress when administering alfalfa saponin extracts to ruminants [98,103]. These findings suggest that saponins might be involved in some cases of frothy bloat, but they are not required for the formation of stable foam in the rumen. Saponin content, ratio between sapogenins and sapogenin structure differ among alfalfa cultivars and might determine the propensity to develop pasture bloat [96,104,105]. Little is known about the effect of environment, season, climatic conditions and growth conditions on saponin content, the ratio between individual sapogenins and on sapogenin structure in alfalfa [105].

A number of factors appear to influence foam strength. Previously, foam strength was found to be maximum around pH 5 to 6 [106,107]. Positively-charged ions (Ca^2+^ and Mg^2+^) may increase foam stability by attracting negatively charged soluble proteins in rumen liquid [66,76,108]. Pectins and simple saccharides are also thought to increase the viscosity of rumen liquid [88,109] while higher fiber content is associated with a lower forage digestibility, a condition which reduces the amount of foaming compounds released [110]. Soluble proteins, small particles and positively charged ions may form a complex at the gas–liquid interface and stabilize ruminal foam [68,75].

## 4. Management Strategies to Reduce Bloat Incidence on Alfalfa Pastures

Grazing management strategies found to reduce the incidence of pasture bloat include allocating cattle to a new pasture in the afternoon instead of the early morning, continuous grazing instead of interrupted grazing, strip grazing to force the animals to graze the whole plants in a short period (lower part of the plant contains less protein and is less digestible) [72,111] and offering alfalfa in an advanced stage of maturity [89]. Even though these grazing management strategies are associated with decreased severity and occurrence of bloat, pasture bloat can occur in the afternoon, with continuous grazing and at full bloom [33].

Anti-bloat agents, which contain oils and alfalfa forage lipids, destabilize foam related to bloat [101,112]. Non-bloating alfalfa forage has a higher lipid (mainly located in the chloroplast) content relative to protein [112]. Anti-foaming agents, like oils and detergents, have been used with success to prevent pasture bloat, but their effectiveness depends on intake, which cannot be guaranteed when supplied as free choice [67,111].

The risk of pasture bloat is negligible when cattle graze pastures with grass. Feeding corn silage or grass hay to cattle before turning them onto alfalfa pasture reduces the incidence of bloat substantially [113,114]. However, beef cattle and sheep are usually not (or not regularly) supplemented during the grazing season. Moreover, in general, grass pastures have a lower nutritional value, lower DM yield and higher fertilization costs compared to alfalfa under North American conditions [33]. Therefore, alfalfa can be grown in mixtures with grass so that livestock can benefit from the positive attributes of both forage families, but even mixtures have limitations. Orchard grass-alfalfa mixtures (25%–50% grass) reduced the incidence of bloat, but did not fully eliminate it [115,116], likely because of plant selection by cattle. Rutter *et al.* [117] found that cattle and sheep grazed on mixed grass-legume pastures (perennial ryegrass/white clover) prefer eating legumes (*ca.* 70%), especially in the morning.

## 5. Selection of Alfalfa with Reduced Protein Degradation

From 1979 onwards, selection at Agriculture and Agri-Food Canada for alfalfa with a low initial rate of degradation was conducted by incubating the top 15 cm of alfalfa plants *in situ* in nylon bags for 4 h in the rumen of fistulated cows. The population of the fourth selection cycle was released as a new cultivar in 1997 under the name AC Grazeland [118]. AC Grazeland harvested at a vegetative stage had a reduced initial rate of degradation, increased NDF and acid detergent fiber content, lower leaf-to-stem ratio; increased leaf epidermis and mesophyll wall thickness compared with the alfalfa cultivar Beaver [118,119,120,121]. In grazing experiments, AC Grazeland at the vegetative stage reduced the incidence and severity of pasture bloat on average by 56% compared to Beaver but it did not fully prevent it [111,118,122]. *In vitro* foam stability was reduced for leaf extract from AC Grazeland compared with other alfalfa cultivars [123]. A similar selection program in Argentina [124] produced a third selection cycle population called Carmina, which reduced the incidence and severity of pasture bloat by 25% [125]. Thus further improvements in protein degradation characteristics are required in order to develop a bloat safe alfalfa cultivar.

A range of methods has been be used for rapid selection of alfalfa samples for different protein properties. The *in vitro* inhibitor method showed a difference of 41, 38 and 31 g/kg·N and 4.2%/h for soluble N, potentially degradable N, ruminal escape protein and degradation rate, respectively, among 19 alfalfa cultivars from different parts of the world while each had a similar total N content [126]. Using the same method, rumen undegradable N differed 21 g/kg·N and N degradation rate differed by 1.9%/h among 27 Canadian cultivars grown in two years and *in vitro* DM digestibility (Tilley and Terry method) of these same cultivars differed by 38 g/kg DM [121]. Protein fractions generated by wet chemical analysis as used in the Cornell Net Carbohydrate and Protein System correlated with *in vitro* inhibitor rumen undegradable CP [127].

Individual amino acids and individual proteins differ in ruminal degradation rate. Thus, protein degradation rates could be decreased by increasing the expression of genes that produce amino acids with a low degradation rate [128,129]. Protein secondary and tertiary structures affect the extent and rate of rumen degradability as well. The number of disulfide (S–S) bonds in the tertiary protein structure was found to affect the extent and rate of protein degradation [130,131]. Sulfur containing amino acids have disulfide bonds, which are less degradable in the rumen. Fourier Transformed Infrared (FTIR) vibration spectroscopy determined that protein with more α-helices relative to β-sheets and protein with less amide I relative to amide II vibration had reduced ruminal protein degradability [132,133]. The previously mentioned AC Grazeland had more α-helices relative to β-sheets and protein with less amide I relative to amide II compared with other cultivars [123] and AC Grazeland tended to have a lower rumen degradability of protein compared with other alfalfas [134]. There was little difference between transgenic alfalfa leaves and their non-transgenic parents in terms of protein molecular structures [123,135], while *in vitro* fermentation characteristics differed [136]. The α-helices:β-sheets ratio decreased and amide I:amide II ratio increased with advancing maturity of alfalfa hay [137]. The α-helices:β-sheets ratio related positively with protein rumen degradability and predicted nutrient supply to cattle while it correlated negatively with amid I:amid II ratio [137], which is opposite from the expectation described above. Further research is required to improve the application of FTIR to predict the protein value of feeds. Twenty-six alfalfa proteins from several genotypes of three alfalfa populations showed a range of degradability using fluorescence two-dimensional gel electrophoresis combined with mass spectrometry [138]. For nine of these proteins, more than 75% remained after 45 min of *in situ* ruminal incubation, while twelve other proteins where more than 50% digested after 45 min, and others were intermediate. After 120 min incubation, 80% remained from four proteins and less than 50% remained from 14 proteins. The main protein (41% of the original sample) with a low degradability after 120 min was the Rubisco large subunit [138]. Previously the Rubisco large subunit was found to have a faster ruminal degradation rate than the Rubisco small subunit [139,140]. Selecting alfalfa plants based on protein structure or protein composition might offer new ways to develop alfalfa with a reduced ruminal degradation rate. Proanthocyanidins are also known to reduce the rate and extent of ruminal protein degradation [9].

## 6. Alfalfa-Proanthocyanidin Containing Forage Mixtures

Proanthocyanidins do not usually accumulate in alfalfa forage [10], but they can be introduced into livestock diets by mixing proanthocyanidin containing forage species into alfalfa pastures [141]. In grazing experiments, mixed alfalfa pastures with 9.1% to 35.5% sainfoin (1.8 to 8.6 g/kg proanthocyanidin) reduced the incidence of bloat as the percentage of sainfoin in the pasture increases. Cattle grazing this mixed pasture had decreased ruminal ammonia concentration, decreased acetate:propionate ratio and decreased ruminal proteolytic activity [141]. However, mixing sainfoin into alfalfa pastures does not fully eliminate pasture bloat [141,142], likely because of competition between plant species (changing the proanthocyanidin content ingested by the animal) and/or plant selection by grazing cattle [141]. Performance of grazing sheep was improved in birdsfoot trefoil/alfalfa mixed pastures (0.8%–1.0% proanthocyanidin) compared with pure alfalfa pastures, but was superior in pure birdsfoot trefoil pastures (3%–6% proanthocyanidin) [143]. Many other beneficial effects on animal health and production were found when grazing proanthocyanidin containing forages like birdsfoot trefoil (Table 4). When forage contains up to 9% proanthocyanidins, approximately 90% of it complexes with host plant constituents (mainly protein) during digestion and only 10% is released as free-proanthocyanidins [144]. Free-proanthocyanidins are required in the mixture to improve the degradation characteristics of non-proanthocyanidin accumulating forage species; its limitation may explain the limited benefit of mixing proanthocyanidin accumulating forages with alfalfa. Proanthocyanidin content of the co-forage should probably be high (>9%) in order to be successful at affecting ruminal degradability from mixtures with alfalfa [9]. It would seem that a problem with grazing mixed pasture is animal selection (palatability of the added forage) that would make it difficult to truly assess the impact of intercropping to control bloat. Therefore it would be beneficial to develop alfalfa that produces moderate amounts of proanthocyanidin in its forage.

## 7. Relation of the Lower Flavonoid Pathway to Stimulate Anthocyanin and Proanthocyanidin

The synthesis of proanthocyanidin in the flavonoid pathway is regulated by many regulatory genes at the transcriptional level. The regulatory genes involved in the flavonoid pathway code for *β*-helix-loop-helix proteins (*βHLH*), *Myb*-like proteins and WD40-like proteins.

In maize (*Zea mays*), several genes regulate its flavonoid pathway. These code for *βHLH* proteins (*Sn*, *B-Peru*, and *Lc*), *Myb*-like proteins (*C1*) and WD40-like proteins (*PAC1*) [158]. Over-expression of the maize *βHLH* gene in birdsfoot trefoil increased the number of proanthocyanidin-containing cells by 50 fold and its leaf concentration was increased by 1% [159]. 

Alfalfa contains proanthocyanidin in the seed coat [160] proving the existence of the flavonoid pathway in alfalfa. Recently, alfalfa was transformed with the *Myb*-like legume anthocyanin-producting gene (*LAP1*) from *Medicago truncatula* [161]. Small but stable amounts of proanthocyanidin-like structures without (−)-epicatechin extension units and multiple glycosylated conjugates of cyanidin were detectable in this deep-purple *LAP1*-transformed alfalfa. In addition, large numbers of regulatory genes involved in the flavonoid pathway were induced [161].

Ray *et al.* [162] transformed alfalfa with three flavonoid pathway regulatory genes of maize (*C1*, *Lc*, and *B-Peru*). Only the *Lc* (*βHLH*) regulatory gene stimulated the accumulation of anthocyanidin under field conditions (97–136 µg/g DM) [163] and anthocyanidin (152 µg/g fresh) and proanthocyanidin (307 µg/g fresh; ~1.5 g/kg DM) after indoor shift to high light intensity [162]. A detailed phytochemical analysis of these genotypes indicated variation in flavone glycosides between the *Lc* (A01-88)-genotypes and in one genotype a two-fold increase in total saponin content (Oleszek and Gruber, AAFC, Saskatoon, Canada; unpublished data). Three of these *Lc*-alfalfa genotypes grown in the field had a reduced initial rate of nitrogen and DM degradation *in vitro* in rumen liquid compared to their non-transgenic parent genotype, but the extent of N and DM degradation was unaffected [163]. These *Lc*-genotypes did not survive the winter in western Canada and therefore three *Lc*-genotypes were crossed, each with different local commercial alfalfa cultivars to be able to do a study over multiple year in the field. As with the parental *Lc*-genotypes, accumulation of extractable and unextractable proanthocyanidin was not detectable in the forage [164,165] while anthocyanidin accumulated in all three years of the study in all three populations ranging from 42.5–349.0 µg/g DM [123,134,136,164], which was a broader and higher range than the 97–136 µg/g DM range for the parental *Lc*-genotypes [163]. Compared with parental non-transgenic alfalfa cultivars, these anthocyanidin accumulating *Lc*-alfalfa crosses had lower protein and higher carbohydrate content [164,165], reduced rate of fermentation and effective degradability [136] higher predicted nutrient availability for animal production [134] and reduced bloat related foaming properties *in vitro* [123]. These suggest that anthocyanidin might also hold promise to improve the nutritive properties of alfalfa.

However, proanthocyanidin concentration in the *LAP1*-alfalfa and *Lc*-alfalfa genotypes was far below levels needed to guarantee bloat safety and to beneficially affect protein metabolism. Hence, further improvements are required to develop an alfalfa cultivar that accumulates a higher amount of proanthocyanidin in its forage. New crosses have been made between *Lc*-alfalfa genotypes (with multiple copies of the *Lc* gene) and *Lc*-alfalfa crossed with LAP1-alfalfa and C1-alfalfa in an attempt to enhance the flavonoid pathway and these new populations are currently under evaluation [166].

## 8. Conclusions

Proanthocyanidin is beneficial in moderate concentrations in temperate legume and ryegrass pastures because of its ability to bind with dietary protein in the rumen, which improves protein utilization and overall animal performance, prevents pasture bloat and reduces environmental impact from pastoral farming systems. However, agronomic performance of proanthocyanidin accumulating legumes is inferior to alfalfa and ryegrass/white clover pastures, which limits their adoption by farmers. Progress is made in enhancing the flavonoid pathway in alfalfa through genetic engineering, but accumulation of proanthocyanidin in the forage remains low to date and public concern might limit adoption of successful cultivars. In addition, improvement in the agronomic performance of proanthocyanidin accumulating legumes is in progress for both monocultures and in mixtures with other forages like alfalfa.

## Figures and Tables

**Table 1:** Composition, tissue disruption and release of nutrients in leaves of several temperate forage legumes.

Item	Legume Species					
Parameter	Alfalfa	White Clover	Red Clover	Birdsfoot Trefoil	Sainfoin	Cicer Milkvetch
*Leaf epidermis + cuticle thickness*						
Upper (µm) ^1^	1.05	1.30	1.04	1.21	1.54	ND
Lower (µm) ^1^	0.93	1.31	0.89	1.22	2.04	ND
Upper (µm) ^2^	21	26	23	30	25	32
*Leaf tissue*						
Cavities air space (%) ^1^	21.2	15.8	10.8	23.8	22.6	ND
Leaf cell wall strength ^3^	weak	weak	mid	strong	mid	Mid
Leaf tissue strength ^3^	low	low	low	low	high	High
Intact leaf mesophyll cells (no.) ^4^	48	7	1	1167	570	811
Microbial tissue disruption (%) ^5^	88	94	89	ND	ND	65
*Release of plant cell constituents*						
Potassium (%) ^6^	57	ND	50	ND	64	ND
Kd conductivity (%/h) ^7^	59	71	75	34	98	ND
Rubisco (%) ^6^	46	ND	16	ND	17	ND
Soluble protein (%) ^6^	24	ND	20	ND	0	ND
Proteolytic activity index ^8^	0.77	0.24	0.17	0.14	ND	ND

ND is not determined. ^1^ Epidermis + cuticle thickness and air space were measured in leaf cross sections by electron microscopy [24]; ^2^ Epidermis + cuticle thickness was measured in leaf sections under a coverslip in 0.55 mM mannitol solution using photographic transparency (shadow graph with stage micrometer scale) [25]; ^3^ Tissue and cell wall strength was determined as chlorophyll released after mechanical disruption by shaking leaves in a tube with glass beads, by a ground glass tissue grinder or by sonication [25]; ^4^ Leaves crushed between two layers of nylon cloth in a mortar with buffer using a pestle. Intact filtrate mesophyll cells were counted with a hemocytometer and light microscope [19]; ^5^ Disappearance of green leaf tissue dry matter after 8 h of ruminal *in situ* incubations [26]; ^6^ Release of plant cell constituent after ingestive mastication [18]; ^7^ Change in conductivity (rate) due to release of minerals from leaves incubated in H_2_O at 39 °C [24]; ^8^ Proteolytic activity of fresh plant tissue incubated over a gelatin substrate gel [27].

**Table 2:** Protein quality characteristics for freeze-dried alfalfa, grass and sainfoin.

Item	RD_DM_^1^	CP	Ruminal CP Degradation			RD_N_:RD_DM_^1^	References
Parameter			Rate	Solubility	Extent		
Unit	(%DM)		(%/h)	(%CP)		(g/kg)	
*Alfalfa*							
Vegetative	65–80	20–27	15–34	40–60	80–90	40–49	[32,44,45,46,47]
Bud	55–77	17–21	15–29	41–47	80–83	34–41	[32,45,47]
Early flower	57–59	17–19	17–31	40–46	75–81	37–41	[32,44,45]
Full flower	58–60	15–16	12–14	41–47	73–79	31–32	[45,47]
Early pod	55–58	16	15–17	52	78–82	36	[46,47]
*Grass*							
Tillering	66–70	20–26	17	32–45	79–82	39–50	[45]
Elongation	52–62	15–21	10–15	27–47	68–82	31–47	[45]
Heading	50–60	12–14	11–12	36–56	70–76	26–30	[45]
Flowering	38–40	7–10	7–11	43–53	61–70	18–27	[45]
*Sainfoin*							
Vegetative	55	16	11.3	16	59	28	[32]
Early flower	48	12	14.1	20	63	26	[32]

^1^ RD_DM_ is rumen degradable dry matter; DM is dry mater; CP is crude protein; RD_N_:RD_DM_ is rumen degradable N:DM ratio.

**Table 3:** Characteristics of cattle prone to pasture bloat compared with non-bloating animals.

**Bloating Cattle**	**References**
Decreased dry matter intake	[71,72]
Decreased saliva production	[73,74]
Increased saliva viscosity	[74]
Decreased clearance rate liquid and particles	[71,75]
**Rumen liquid of bloating cattle**	**References**
Decreased Na^+^	[76]
Increased Ca^2+^, Mg^2+^ and K^+^	[76]
Increased viscosity	[77,78]
Increased ethanol precipitated film with higher CP content	[79,80]
Increased buoyancy of particle matter	[71,75]
Increased small particle retention	[71,75,81]
Increased foam volume and stability	[64,65]
Similar protein concentration	[75,81]

**Table 4:** Effect of feeding birdsfoot trefoil and big trefoil with proanthocyanidin concentrations between 2% and 5% on performance of sheep and cattle.

Trait	References
*Sheep*	
Increased wool growth	[145,146,147,148]
Increased milk yield	[146]
Increased ovulation rate/number lambs born	[148,149]
Increased lamb weight gain	[147,150]
Reduced intestinal parasite load	[148,151]
*Cattle*	
Increased milk production	[152,153,154,155]
Increased milk protein production	[154,155]
Decreased milk fat production	[155]
Reduced milk saturated fatty acids	[156]
Increased milk ω-3 fatty acids	[156]
Increased weight gain	[157]
